# Peptide binding to cleaved CD31 dampens ischemia/reperfusion-induced intestinal injury

**DOI:** 10.1186/s40635-018-0192-3

**Published:** 2018-08-15

**Authors:** Quoc Thang Hoang, Alexandre Nuzzo, Liliane Louedec, Sandrine Delbosc, Francesco Andreata, Jamila Khallou-Laschet, Maksud Assadi, Philippe Montravers, Dan Longrois, Olivier Corcos, Giuseppina Caligiuri, Antonino Nicoletti, Jean-Baptiste Michel, Alexy Tran-Dinh

**Affiliations:** 1 INSERM LVTS U1148, Paris-Diderot, Université Sorbonne, Paris, France; 2 Department of Anesthesiology and Surgical Critical Care, Pham Ngoc Thach University of Medicine, Ho Chi Minh City, Vietnam; 3 Structure d’URgences Vasculaires Intestinales (SURVI), CHU Beaujon, Clichy, France; 4 Département d’anesthésie-réanimation, CHU Bichat-Claude Bernard, 46, rue Henri Huchard, 75877 Paris Cedex 18, France; 5 INSERM UMR 1152, Paris-Diderot, Université Sorbonne, Paris, France

## Abstract

**Background:**

CD31 is a key transmembrane neutrophil immunoregulatory receptor. Mesenteric ischemia/reperfusion-induced neutrophil activation leads to a massive cleavage and shedding of the most extracellular domains of CD31 into plasma, enhancing the deleterious effect of neutrophil activation. We have evaluated the preventive therapeutic potential of an engineered synthetic octapeptide (P8RI), which restores the inhibitory intracellular signaling of cleaved CD31, in an experimental model of acute mesenteric ischemia/reperfusion.

**Methods:**

In a randomized, controlled, and experimenter-blinded preclinical study, mesenteric ischemia/reperfusion (I/R) was induced in Wistar rats by superior mesenteric artery occlusion for 30 min followed by 4 h of reperfusion. Three groups of rats were compared: I/R + saline perfusion (I/R controls group, *n* = 7), I/R + preventive P8RI perfusion (P8RI group, *n* = 7), and sham-operated rats + saline perfusion (sham group, *n* = 7).

**Results:**

Compared with I/R controls, P8RI perfusion significantly decreased intestinal ischemia/reperfusion injury (Chiu’s score, *P* = 0.01; epithelial area, *P* = 0.001) and bacterial translocation (plasma *Escherichia coli* DNA, *P* = 0.04) and could limit intestinal bleeding (*P* = 0.09). P8RI decreased neutrophil activation assessed by matrix metalloproteinase-9 release in plasma (*P* < 0.001) and in the intestinal wall, albeit without statistical significance (*P* = 0.06 and *P* = 0.058 for myeloperoxydase). Inhibition of CD31 cleavage from neutrophils could play a major role in the protective effects of P8RI (*P* < 0.0001).

**Conclusions:**

Preventive administration of P8RI, a CD31-agonist peptide, could decrease I/R-induced intestinal injury by potentially limiting neutrophil activation.

**Electronic supplementary material:**

The online version of this article (10.1186/s40635-018-0192-3) contains supplementary material, which is available to authorized users.

## Background

Acute mesenteric ischemia is a life-threatening emergency with a high mortality rate, reaching 58% in intensive care unit patients [1]. It can lead to an overwhelming inflammatory response [2], bacterial translocation [3], digestive bleeding, intestinal necrosis, multiple organ failure [4], and death. There is no specific therapy targeting intestinal ischemia/reperfusion injury.

Neutrophils are key players in this pathology [5, 6]. As in all leukocytes, the activation of neutrophils is tightly controlled by inhibitory co-receptors. Among the inhibitory receptors expressed by neutrophils, CD31 is a major target. Indeed, CD31 is a key leukocyte immunoregulatory receptor [7], and neutrophils are among the cells that express the highest number of CD31 molecules [8].

CD31 is a 130-kDa transmembrane glycoprotein receptor also expressed by the other leukocytes, platelets, and endothelial cells. Transhomophilic engagement of CD31 raises the activation threshold of these cells via activation of SH2 tyrosine phosphatase [9]. When submitted to activating stimuli strong enough to overcome the activation threshold, cell activation is accompanied by the cleavage and shedding into plasma of most of the extracellular CD31, thereby interrupting the downstream cell signaling and leaving cell activation unharnessed [7]. It is of interest that after shedding, a short portion of the membrane-proximal extracellular sequence remains exposed at the cell surface. We recently engineered a drug-suitable synthetic octapeptide (termed P8RI), which is able to bind to this short portion and to restore the inhibitory intracellular signaling of cleaved CD31 [10, 11], including in inflammatory conditions linked to ischemia-reperfusion injury [12]. This peptide is derived from a parent sequence that has previously been successful in models of acute vascular inflammatory conditions [13]. Herein, we have evaluated the therapeutic potential of P8RI, via a putative protective effect on neutrophil activation, in an experimental model of acute mesenteric ischemia/reperfusion.

## Methods

Procedures and animal care complied with principles formulated by the National Society for Medical Research (animal facility agreement: n° B75-18-03, experimentation authorization n° 75-101, APAFiS#8724). Eight-week-old male Wistar rats weighing 280 g were purchased from Janvier laboratory.

### Model of acute mesenteric ischemia/reperfusion

A total of 28 rats were randomized into four groups: an ischemia/reperfusion (I/R) group treated with saline perfusion (I/R controls, 7 rats), an ischemia/reperfusion group treated with P8RI perfusion (P8RI group, 7 rats), and a sham-operated group (sham group, 7 rats). A group of sham-operated rats treated by P8RI perfusion (sham-P8RI group, 7 rats) was added in order to assess for potential histological changes within the normal bowel epithelium in response to P8RI.

Rat body temperature was maintained at 37.5 °C by a heated surgical table. Non-fasting rats were anesthetized by intraperitoneal injection of 2 mg/kg of urethane, and analgesia was performed by subcutaneous injection of buprenorphine (0.05 mg/kg). Cannulation of the right jugular vein and right carotid artery were performed for perfusion, blood sampling, and arterial pressure measurements. After laparotomy, the superior mesenteric artery (SMA) was exposed but not occluded in the sham group. In the control and P8RI groups, the SMA was clamped. The clamp was removed after 30 min of ischemia, followed by 4 h of reperfusion. A single 3.5-mg/kg intravenous bolus of P8RI was administered 5 min before clamping SMA and was followed by a continuous perfusion at 3.5 mg/kg/h until the end of reperfusion. The same procedure was performed for the control group, except for the perfusion of P8RI, which was substituted by an equivalent volume of saline perfusion. The experimenter was blinded with regard to the treatment administered. Arterial blood pressure and diuresis were monitored throughout the experiment. Blood and peritoneal fluid were sampled in EDTA tubes before the laparotomy and 30 min after clamping the SMA. Blood samples were also collected every hour during the reperfusion, and peritoneal fluid samples after 2 and 4 h of reperfusion. Samples were centrifuged for 30 min at 16,000*g*. Supernatants were used for assays. At the end of the procedure, a total enterectomy was performed after rats were euthanized. The intestinal luminal content was collected for the quantification of intestinal bleeding. Histological analysis was performed on a central section of 2-cm-long jejunum specimen as previously recommended [14]. The remaining small bowel was homogenized. Homogenates were centrifuged for 30 min at 16,000*g*, and supernatants were used for assays.

### Assessment of mesenteric ischemia/reperfusion-induced intestinal injury

#### Histological analysis of the intestinal mucosa

Highly glycosylated mucins and nuclei were stained respectively with Alcian Blue and nuclear fast red on transverse paraffin-embedded sections of the small intestine. Histological evaluation was performed using the grading system described by Chiu et al. [15], as described in Additional file 1 (Table S1).

#### Morphometric analysis of the small bowel

A morphometric evaluation of histological gut sections was performed using QWin software to determine the luminal area, epithelial and muscular layer areas, and the overall surface area, as described in Additional file 1 (Figure S1).

#### Intestinal bleeding

The intestinal bleeding was assessed by the quantification of heme concentration in the small intestine luminal content using formic acid, as described in Additional file 1.

#### Neutrophil activation in the small bowel tissue

Neutrophil activation was assessed by the intestinal tissue concentrations of matrix metalloproteinase-9 (MMP-9) and myeloperoxydase (MPO), using enzyme-linked immunosorbent assays, as described in Additional file 1.

### Assessment of mesenteric ischemia/reperfusion-induced neutrophil activation in plasma

Neutrophil activation in plasma was assessed by the plasma concentration of MMP-9 using an enzyme-linked immunosorbent assay, as described in Additional file 1.

### Assessment of mesenteric ischemia/reperfusion-induced bacterial translocation

Bacterial translocation was assessed by the quantification of plasma DNA from *Escherichia coli* at 4 h of reperfusion after the onset of mesenteric ischemia, using a real-time PCR technique, as described in Additional file 1.

### Assessment of soluble CD31 in plasma

The shedding of CD31 into plasma was assessed by measuring soluble plasma CD31 at repeated time points before, during ischemia, and during the reperfusion period. Within this timeframe, raised levels of CD31 can only derive from the cleavage and shedding of the membrane-anchored molecule. The assay was based on the use of cytometric polystyrene beads. A polyclonal antibody targeting rat CD31 (R&D, #AF3628) was immobilized onto COOH-magnetic beads (MC10035-01, BioRad). CD31 capture beads were then incubated with the EDTA-plasma (diluted 1:6) from individual rats and each time point for 90 min at room temperature. After repeated washing, the soluble circulating CD31 that was captured by the beads was revealed with using a phycoerythrin-labeled monoclonal antibody (clone TLD-3A12) directed to the most membrane-distal portion of rat CD31. Median fluorescent intensity was analyzed from a total of 100 beads per sample on a BioPlex 200® System (BioRad). A standard curve was obtained using serial dilutions of recombinant CD31.

### Sample size calculation

The study was designed with 90% power to detect a relative 50% difference in epithelial layer area between I/R controls and P8RI group. Statistical testing was performed at the two-tailed level of 0.05 using a *t* test. Based on preliminary data indicating that mean epithelial layer area after mesenteric ischemia/reperfusion was 100 μm^2^ (SD ± 27), sample size calculation indicated seven rats per group.

### Statistical analysis

Quantitative data are expressed as medians with interquartile range (IQR) or means ± SEM. Mann-Whitney U test, two-way ANOVA with post hoc multiple comparison Bonferroni tests and Spearman correlation were performed as appropriate. Principal component analysis was performed to study how I/R conditions (controls, P8RI-treated, and sham-operated) impact the relationships (correlations) between the epithelial area, neutrophil activation, CD31 cleavage, intestinal bleeding, intestinal tissue injury, and bacterial translocation.

## Results

The data that support the findings of this study are available from the corresponding author upon reasonable request.

### Effect of P8RI on mesenteric ischemia/reperfusion-induced intestinal injury

#### Histological analysis of the intestinal mucosa

**Fig. 1:**
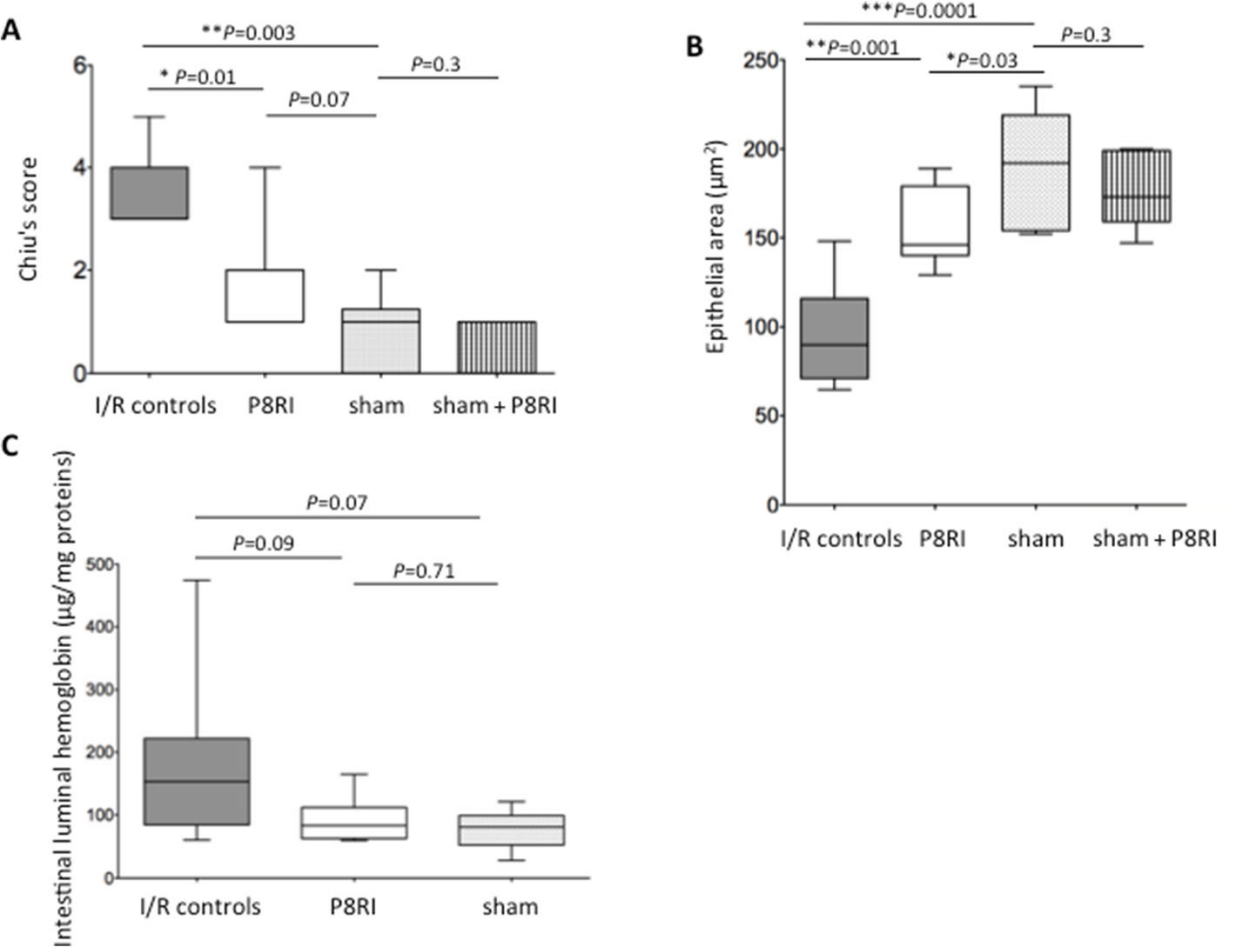
Effect of P8RI on mesenteric ischemia/reperfusion-induced intestinal injury. Ischemia/reperfusion-induced intestinal injury was assessed by histological analysis using Chiu’s score that ranges from a normal mucosa (score = 0) to a total mucosal necrosis (score = 6), and morphometric analysis using Qwin software from scanned histological sections by a nanozoomer in I/R controls, P8RI, and sham groups. **a** The Chiu’s score of intestinal injury was lower in the P8RI group compared to I/R controls (*P = 0.01). **b** The epithelial area was higher in the P8RI group than in I/R controls (**P = 0.001). **c** The intestinal luminal hemoglobin concentration only tended to be lower in the P8RI group than in I/R controls, without reaching statistical significance (*P* = 0.09)

I/R greatly increased the intestinal mucosa injury as assessed by Chiu’s score compared to sham-operated rats (*P* = 0.003), and P8RI administration significantly limited this injury compared to saline injection (*P* = 0.01). Indeed, Chiu’s score was not significantly different between the P8RI and sham groups (*P* = 0.07), and between sham-operated rats perfused with either saline or P8RI (*P* = 0.3) (Fig. 1a). Typical histological sections of the small intestine for the four groups of rats are provided in Additional file 1 (Figure S2).

#### Morphometric analysis of the small bowel

Morphometric analysis confirmed histological results by showing that I/R markedly decreased the small bowel epithelial area compared to sham-operated rats (*P* = 0.0001). P8RI perfusion limited the abrasion of the epithelial area compared to saline injection (*P* = 0.001), but it remained significantly lower than in the sham group (*P* = 0.03). The epithelial area was not different in sham-operated rats treated with P8RI perfusion compared to those perfused with saline (*P* = 0.3) (Fig. 1b). The epithelial area was negatively correlated to Chiu’s score (*r* = − 0.72, *p* = 0.0002).

I/R also increased smooth muscle layer injury since morphometric analysis showed that its area was decreased compared to the sham group (*P* = 0.0008). P8RI perfusion failed to limit smooth muscle layer thinning (*P* = 0.35). Indeed, the smooth muscle area remained significantly lower in the P8RI group than in the sham group (*P* = 0.002).

#### Intestinal bleeding

I/R led to more intestinal bleeding, as assessed by the luminal concentration of hemoglobin, which tended to be higher than in sham-operated rats, although it was not statistically significant (*P* = 0.07). P8RI administration could limit the bleeding as the hemoglobin concentration in the treated group tended to be lower than in I/R controls, despite it was not statistically significant (*P* = 0.09) (Fig. 1c). Intestinal bleeding was not different between the P8RI and sham group (*P* = 0.71).

### Effect of P8RI on mesenteric ischemia/reperfusion-induced neutrophil activation

#### Neutrophil activation in the small bowel tissue

**Fig. 2:**
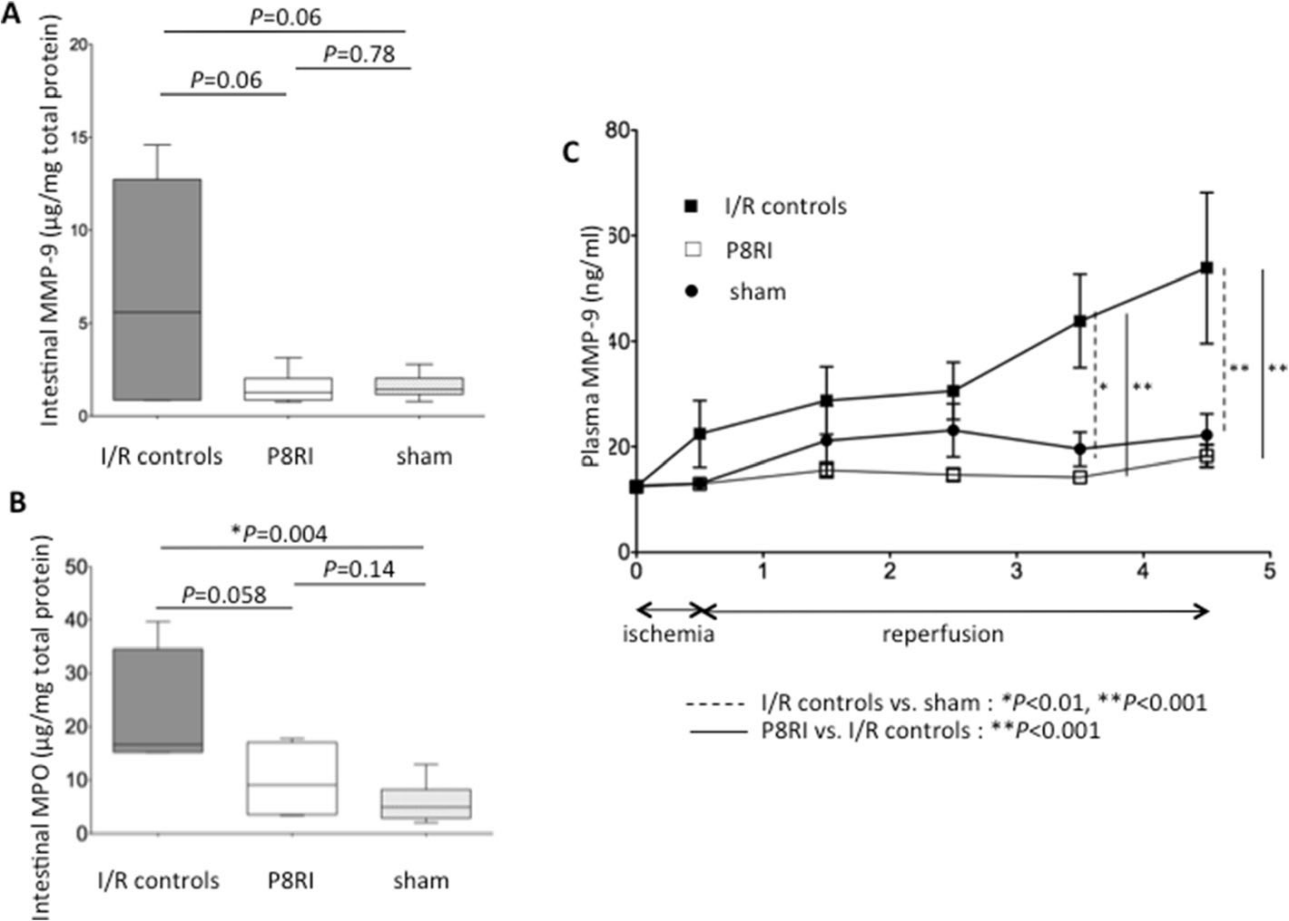
Effect of P8RI on mesenteric ischemia/reperfusion-induced neutrophil activation. Mesenteric ischemia/reperfusion-induced neutrophil activation was assessed by the detection of MMP-9 and MPO release in the small bowel tissue and plasma. **a**, **b** The concentrations of MMP-9 and MPO in the intestinal tissue were almost significantly lower in the P8RI group than in I/R controls (*P* = 0.06 and *P* = 0.058, respectively). **c** The plasma concentration of MMP-9 was lower in the P8RI group than in I/R controls at 3 h (**P < 0.001) and 4 h (**P < 0.001) of reperfusion after mesenteric ischemia. MMP-9: matrix metalloproteinase-9

In I/R controls, intestinal tissue concentration of MMP-9 and MPO respectively tended to be higher (*P* = 0.06) and was significantly higher (*P* = 0.004) than in sham-operated rats. P8RI-treated rats presented almost significant lower MMP-9 (*P* = 0.06) and MPO (*P* = 0.058) levels than I/R controls. P8RI group had similar levels to those of the sham group (*P* = 0.78 for MMP-9 and *P* = 0.14 for MPO) (Fig. 2a, b).

#### Neutrophil activation in plasma

Before laparotomy, the plasma concentration of MMP-9 was similar in the three groups. We found a significant effect of the treatment (*P* = 0.0005) and time (*P* < 0.0001) on the plasma concentration of MMP-9. Plasma neutrophil activation assessed by the plasma concentration of MMP-9 was higher in the I/R controls than in the sham group at 3 h (*P* < 0.01) and 4 h (*P* < 0.001) of reperfusion. P8RI administration limited plasma MMP-9 compared to saline injection at 3 h (*P* < 0.001) and 4 h (*P* < 0.001) of reperfusion. Plasma neutrophil activation was not significantly different between the P8RI and sham groups (*P* > 0.05 after ischemia and after each time of reperfusion) (Fig. 2b).

### Effect of P8RI on mesenteric ischemia/reperfusion-induced bacterial translocation

**Fig. 3:**
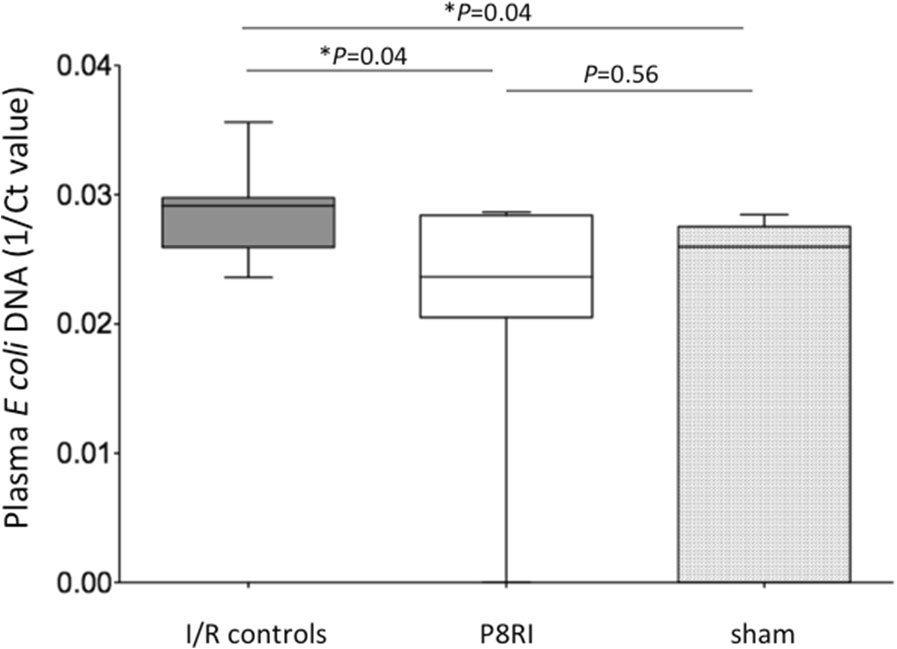
Effect of P8RI on mesenteric ischemia/reperfusion-induced bacterial translocation. Bacterial translocation was assessed by the quantification of plasma DNA from *Escherichia coli* using real time PCR. Ct value represents the minimum number of cycles to detect plasma *Escherichia coli* DNA and is inversely correlated to the quantity of plasma DNA from *Escherichia coli*. P8RI administration limited the plasma concentration of DNA from *E*. *coli* compared to saline injection (*P = 0.04)

Four hours of reperfusion after 30 min of ischemia increased the plasma concentration of DNA from *Escherichia coli* (1/Ct value) compared to sham-operated rats (*P* = 0.04). P8RI administration limited the bacterial translocation compared to saline injection (*P* = 0.04) providing levels not significantly different from those of the sham group (*P* = 0.56) (Fig. 3).

### Effect of P8RI on mesenteric ischemia/reperfusion-induced shedding of CD31 into plasma

**Fig. 4:**
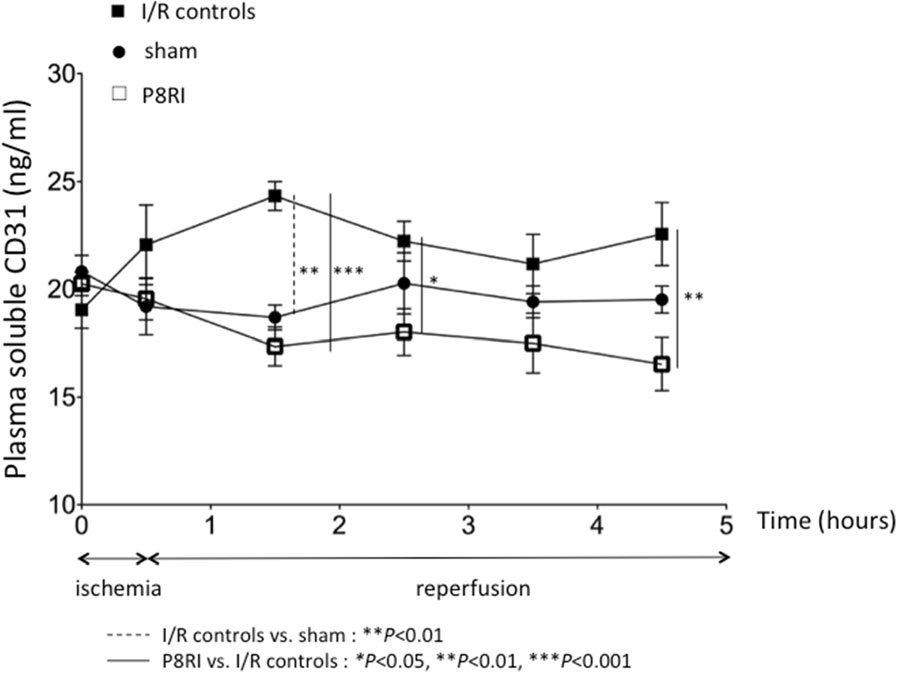
Effect of P8RI on mesenteric ischemia/reperfusion-induced shedding of CD31 into plasma. The shedding of CD31 into plasma was assessed by the detection in plasma of the soluble CD31. The concentration of soluble CD31 in plasma was lower in the P8RI group than in I/R controls at 1 h (***P < 0.001), 2 h (*P < 0.05), and 4 h (**P < 0.01) of reperfusion after mesenteric ischemia

Before laparotomy, the shedding of CD31 into plasma concentration was similar in the three groups. We found a significant overall effect of the treatment (*P* < 0.0001) but not of time (*P* = 0.93) on this parameter. The shedding of CD31 into plasma increased after 1 h of reperfusion following mesenteric ischemia compared to sham-operated rats (*P* < 0.01). P8RI perfusion significantly limited this process at 1 h (*P* < 0.001), 2 h (*P* < 0.05), and 4 h (*P* < 0.01) of reperfusion compared to saline injection. The shedding of CD31 into plasma was not significantly different between the P8RI and sham groups after ischemia and after each reperfusion time point (Fig. 4).

### Effect of P8RI on neutrophil activation, CD31 cleavage, intestinal bleeding, cell death, bacterial translocation, and epithelial injury induced by mesenteric ischemia/reperfusion

**Fig. 5:**
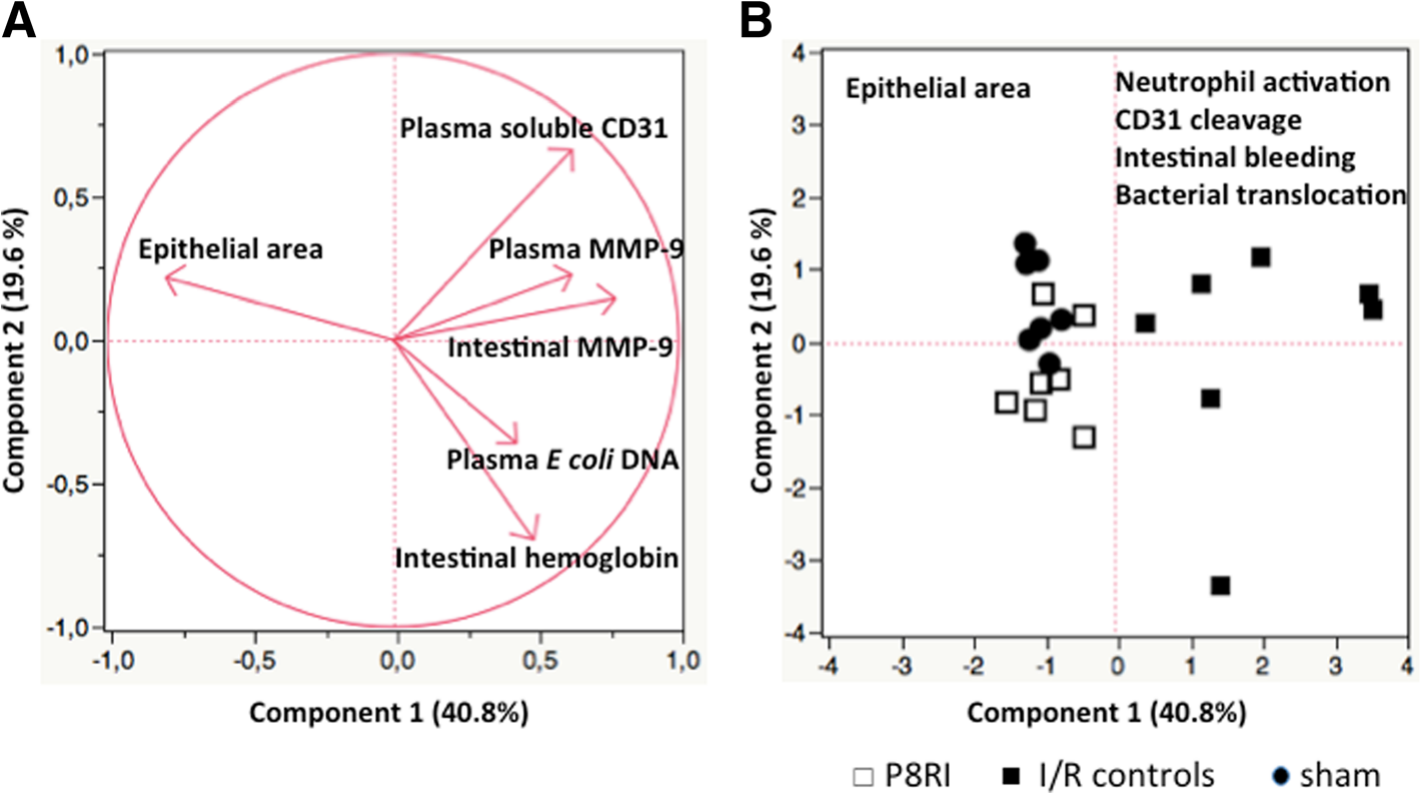
Role of CD31 in neutrophil activation and intestinal injury after acute mesenteric ischemia. **a** Principal component analysis showed positive correlations between intestinal MMP-9, plasma MMP-9, intestinal luminal hemoglobin, plasma *E.* coli DNA, and the shedding of CD31 into plasma in all groups. Conversely, epithelial area was strongly negatively correlated to these parameters. **b** Principal component analysis identified a clustering of the P8RI and sham groups, widely separated from I/R controls. MMP-9: matrix metalloproteinase-9

In P8RI, I/R controls, and sham groups, principal component analysis showed a positive correlation between intestinal MMP-9, intestinal luminal hemoglobin, plasma *E. coli* DNA, and the shedding of CD31 into plasma. On the contrary, epithelial area was strongly negatively correlated to these parameters (Fig. 5a). Principal component analysis allowed the identification of a clustering of the sham and P8RI-treated groups, which were widely separated from I/R controls (Fig. 5b). This result illustrates the major role of CD31 cleavage in neutrophil activation and I/R-induced intestinal injury and may explain the protective effect of P8RI, which restores the immunoregulatory functions of cleaved CD31.

## Discussion

In this study, we showed a protective effect of the preventive administration of P8RI in an experimental model of acute mesenteric ischemia/reperfusion. P8RI is a CD31-agonist peptide that restores the inhibitory function of cleaved CD31 resulting from the activation of leukocytes, endothelial cells, and platelets. Thereby, P8RI reinstates key functions of CD31 in vascular homeostasis [16].

P8RI perfusion protected the intestinal mucosa from ischemia/reperfusion-induced injury as we observed a significant limitation in the abrasion of the villi and capillaries. This effect was potentially mediated by the decrease in neutrophil activation, as assessed by MMP-9 and MPO release into the small bowel tissue and bloodstream. Rosario et al. have shown that the higher protease activity in the intestinal wall after a mesenteric ischemia/reperfusion episode originated from the infiltration of neutrophils and the release of MMP-9 [17]. MMP-9 is the main protease stored in the tertiary granules of neutrophils, more readily mobilized under low chemotactic stimulation levels [18]. In addition, MMP9 is highly diffusible from the tissue to plasma [19]. MMP-9 activation is crucially involved in ischemia/reperfusion injury by modulating extracellular matrix turnover, by triggering inflammatory cell migration and pro-inflammatory mediator release and the stimulation of angiogenesis [20]. Besides, tissue-associated MPO release was shown to be a reliable index of neutrophil infiltration in the intestinal mucosa [21]. In contrast to the intestinal mucosa, we observed no protection by P8RI perfusion against muscular layer injury by P8RI perfusion. However, it has been shown that the neutrophil infiltration in the intestinal mucosa begins as early as 1 h after ischemia/reperfusion, whereas it is delayed by 24 to 72 h in the muscular layer [22]. This could explain why we failed to detect a protective effect on the muscular layer at 4 h of reperfusion after 30 min of ischemia.

A basal circulating form of CD31 was detected in I/R controls, P8RI, and sham groups. It has been shown that, in normal human plasma, there exists a soluble form of mature CD31 containing the cytoplasmic tail and reaching a concentration of 10 to 25 ng/ml, which is secreted only 48 h after intracellular synthesis [23]. Thus, the soluble CD31 quantified here was more likely to result from the cleavage and shedding of CD31 after ischemia/reperfusion than to represent the mature form.

The cleavage of CD31 strongly correlated with the epithelial layer injury, neutrophil activation, and cell death. It emphasizes the important role of CD31 in maintaining the integrity of intestinal mucosa. The protective effect of P8RI could be related to the inhibition of CD31 cleavage from neutrophils, thus decreasing their protease activity on the intestinal mucosa.

Bacterial translocation is a specific feature reflecting the loss of epithelial cell integrity after mesenteric ischemia/reperfusion and is a trigger of the systemic innate immune response and sepsis [24]. We found that P8RI decreased bacterial translocation as assessed by the detection of DNA from *Escherichia coli* in plasma using a real-time PCR technique, a more sensitive assay than bacterial blood cultures [25].

Finally, the absence of any effect of P8RI in sham-operated rats was expected, since this peptide is intended to restore the function of a constitutive physiological receptor. As shown by previous work, CD31 agonist peptide does not trigger CD31 signaling in resting cells at the surface of which an intact CD31, unable to bind the peptide, is expressed [26].

This study has several limitations. First, P8RI has not been tested for curative treatment, but it was useful to assess its protective role on a “controlled” mesenteric ischemia/reperfusion, a clinical condition encountered in repair of the descending aorta [27]. Future studies are needed to specifically assess the therapeutic potential of P8RI in curative experiments, with the treatment starting after the occurrence of acute mesenteric ischemia. Second, the cell type responsible for the shed CD31 in plasma was not determined. It could originate from tissue and/or blood neutrophils, and/or endothelial cells and/or platelets. In humans, the extracellular domain of CD31 is shed at different sites depending on the cellular origin (neutrophils, platelets, or endothelial cells) and released in the circulation as a soluble form of CD31 [28, 29]. Our laboratory has developed a cytometric bead array technology to identify and quantify different soluble fractions of shed CD31 according to their cellular origin [30, 31]. Unfortunately, this technology is not available for use in rats. Instead, we used a polyclonal antibody that targeted all forms of shed CD31 in plasma. Finally, we could not clearly demonstrate that the protective effects of P8RI on I/R-induced intestinal injury derived from the limitation of neutrophil activation. If cell-specific CD31 knockout mice could be available, specific experiments could be performed in order to directly assess the putative neutrophil-mediated effect of P8RI in intestinal I/R injury.

## Conclusions

In conclusion, our data shows that a peptide able to bind to truncated membrane-anchored CD31, such as P8RI, holds a promising therapeutic potential to reduce the intestinal damage inflicted by I/R. The action of the peptide is likely exerted on cells of the innate immunity, which are in the first line of defense against acute tissue injury. Notably, all the cells of the innate immune system express high levels of CD31 [8], and the most abundant population of such cells is represented by neutrophils. Altogether, the present work suggests that P8RI could reduce the extent of intestinal I/R injury by modulating both the local and systemic response of neutrophils.

## Additional file

Additional file 1: **Table S1.** Histological grading system: Chiu's score. **Figure S2.** Histological sections of the small intestine. **Figure S2.** Histological sections of the small intestine. (DOCX 480 kb)

## Abbreviations

I/R: Ischemia/reperfusion
MMP-9: Matrix metalloproteinase-9
SMA: Superior mesenteric artery
