# Supplementary material for: Peptide binding to cleaved CD31 dampens ischemia/reperfusion-induced intestinal injury
Source: Intensive Care Med Exp. 2018 Aug 15;6:27. doi: 10.1186/s40635-018-0192-3 (PMC6093833; doi:10.1186/s40635-018-0192-3)
Supplement: Supplementary file 1 — Table S1. Histological grading system: Chiu's score. Figure S2. Histological sections of the small intestine. Figure S2. Histological sections of the small intestine. (DOCX 480 kb) [file 40635_2018_192_MOESM1_ESM.docx]

**Additional file 1**

**1) Materials and methods**

- **Table S1: Histological grading system: Chiu's score**

| Grade | Histological appearance |
| --- | --- |
| 0  1  2  3  4  5 | Normal  Subepithelial oedema, partial separation of apical cells  Epithelial cell sloughing from tips of villi  Progression of sloughing to base of villi  Partial mucosal necrosis of lamina propria  Total mucosal necrosis |

- **Figure S1: Morphometric analysis of the intestinal wall.**

Histological gut sections were scanned (slide scanner, Hamamatsu Nanozoomer) (A) and analysed using a custom QWin software (B). The luminal area (in blue) was defined by thresholding the space inside the intestinal ring whose external border was limited by the epithelium. The muscular layer (in red) was defined as extending from the basal side of the epithelial layer to the external border of the intestinal ring. The epithelial layer (in green) was defined as the area contained between the lumen and muscular layer.


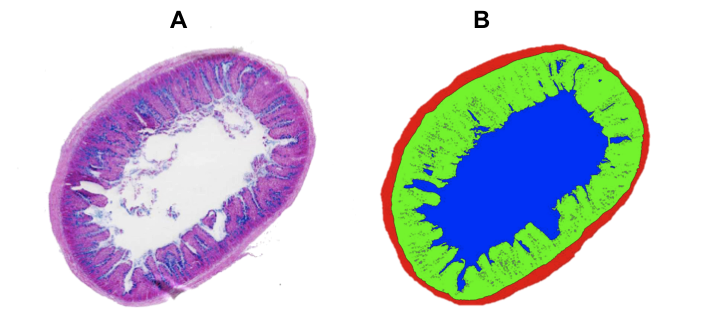


- **Figure S2: Histological sections of the small intestine**

Typical histological sections of the small intestine for the 4 groups of rats.


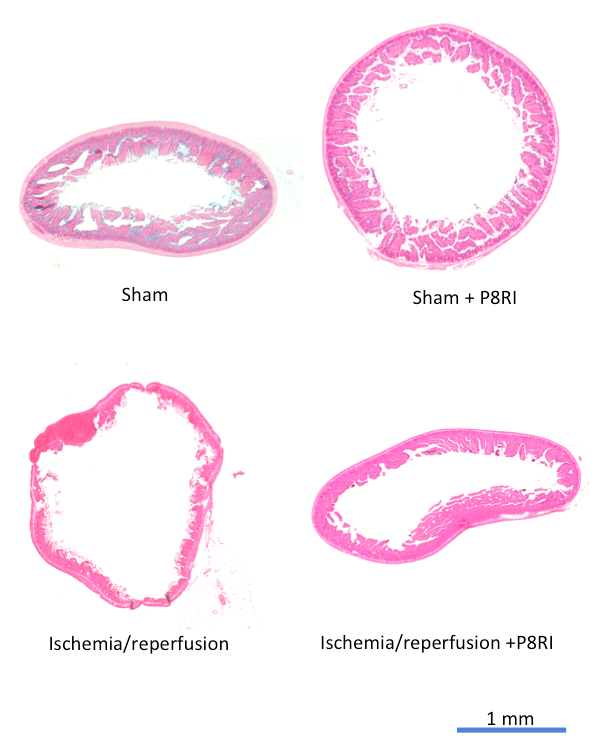


- **Assessment of intestinal bleeding**

The degree of intestinal bleeding was assessed by quantification of the heme content in the luminal content of the small intestine. This luminal content was homogenized in distilled water and centrifuged for 30min at 16,000g. The heme content, used as a surrogate estimation of the luminal hemoglobin, was assessed by addition of formic acid to the supernatant and monitoring the optical density at a wavelength of 405nm (33). The heme content was normalized to the total protein concentration of the luminal content measured by colorimetric protein quantification (BCA protein assay kit, Sigma).

- **Assessment of neutrophil activation in the small bowel tissue.**

The intestinal tissue was homogenized (TissuLyser, Quiagen) and extracted using RIPA buffer. The extract was centrifuged for 30min at 16,000g and the supernatant was used for assays. Total protein concentration in the supernatant was measured using a BCA protein assay kit (Sigma). Intestinal matrix metalloproteinase-9 (MMP-9) and myeloperoxydase (MPO) were used as a marker of intestinal tissue neutrophil activation. The intestinal tissue concentration of MMP-9 and MPO were determined using an enzyme-linked immunosorbent assay for rat (rat total MMP-9 DuoSet ELISA kit, R&D systems and MPO, Rat, ELISA kit Hycult Biotech). The capture antibody was coated on 96-well half area microplates. A blocking buffer solution was used to block the remaining protein binding sites before incubating the supernatant obtained from centrifugation of the small bowel homogenate. The detection antibody was adjusted for the double antibody sandwich enzyme-linked immunosorbent assay protocol. Streptavidine and color reagent were used for the detection of the antigen-antibody reaction. The absorbance was analyzed at a wavelength of 450nm using a Tecan monochromator plate reader.

- **Assessment of bacterial translocation**

Bacterial translocation was assessed by the quantification of genomic DNA from *Escherichia coli* in blood. The technique used real time PCR (CFX96^TM^ Real Time System, BioRad). DNA extractions were performed using 20μL of a dilution buffer and 0.5μL of a DNA release additive (Phire Tissu Direct PCR Master Mix, Thermo). After incubation at room temperature (5min), plasma was denatured at 98^°^C (2min) and centrifuged at 16,000 g (5min). One microliter of the supernatant was used to perform a real-time PCR with *Escherichia coli’s specific* primers (Eurogentec). The amplification was programmed for a first cycle at 50°C (2min), a second cycle at 95°C (15min), 50 cycles at 95°C (40sec) and a last cycle at 60°C (1min). The Ct value represented the number of cycles at which a sufficient quantity of amplified DNA had accumulated to yield a detectable fluorescence signal in plasma. The plasma concentration of *Escherichia coli* DNA was proportional to the inverse of the Ct value (1/Ct value).
